# Correction: Effects of structured exercise programmes on physiological and psychological outcomes in adults with inflammatory bowel disease (IBD): A systematic review and meta-analysis

**DOI:** 10.1371/journal.pone.0307509

**Published:** 2024-07-16

**Authors:** Katherine Jones, Rachel Kimble, Katherine Baker, Garry A. Tew

Fig 1 is uploaded incorrectly. Please see the correct Fig 1 here.

**Fig 1:**
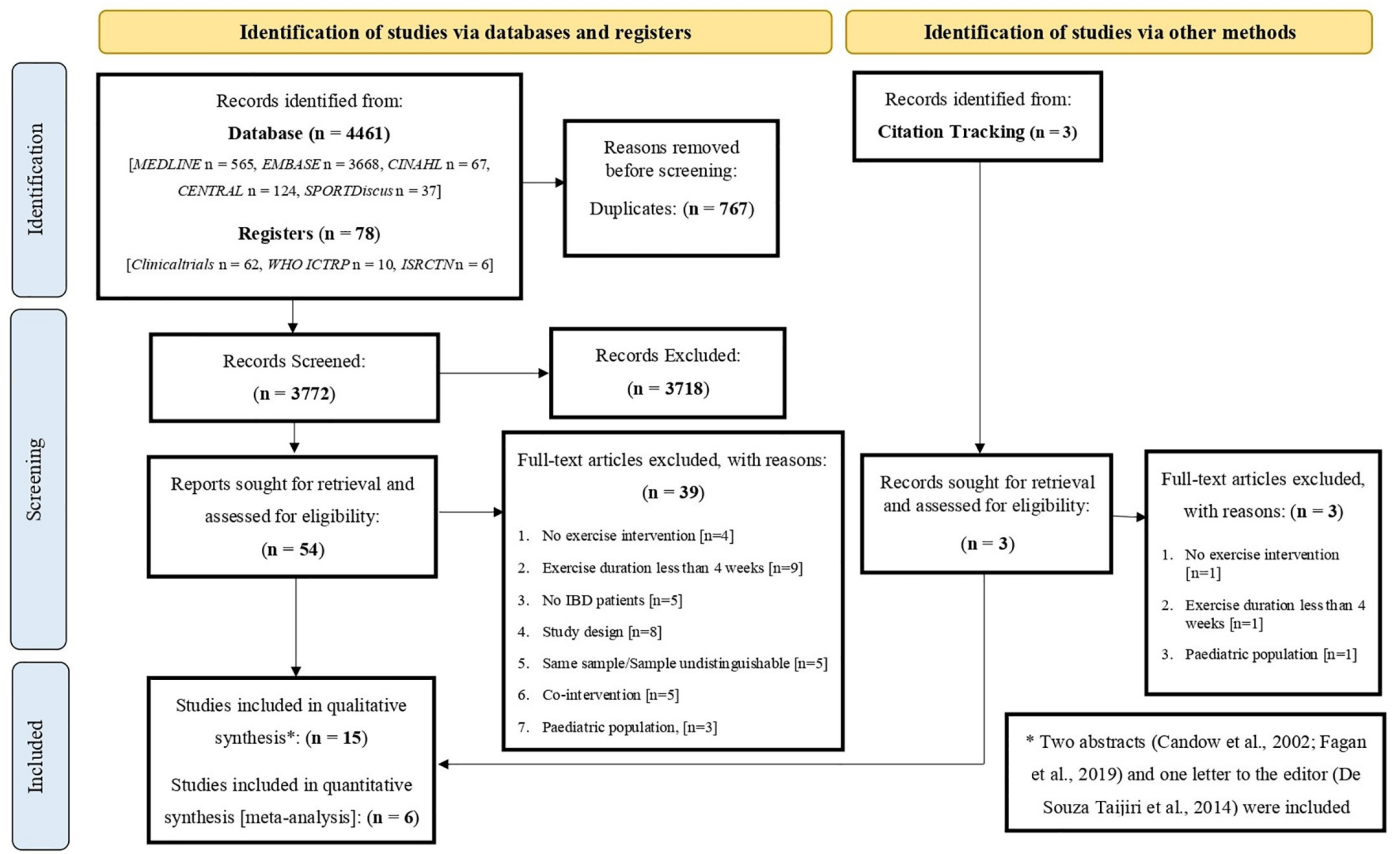
PRISMA flow diagram of literature search and study selection phases. n, number; CENTRAL, Cochrane Central Register of Controlled Trials; WHO ICTRP, World Health Organisation International Clinical Trials Registry Platform.
