# Supplementary material for: Effects of structured exercise programmes on physiological and psychological outcomes in adults with inflammatory bowel disease (IBD): A systematic review and meta-analysis
Source: PLoS One. 2022 Dec 1;17(12):e0278480. doi: 10.1371/journal.pone.0278480 (PMC9714897; doi:10.1371/journal.pone.0278480)
Supplement: S1 Table — (DOCX) [file pone.0278480.s002.docx]

**S1 Table.** Deviations from registered protocol

| **Protocol Method** | **Deviation from protocol method, with justification** |
| --- | --- |
| Planned to include RCTs only | Initial reviewing of including only RCTs failed to identify many studies of interest. Following the search non-randomised controlled trials and observational studies were included as another study of inclusion as a means to expand the volume of research evidence. To comply to this change, the risk of bias tool ROBINS-I was added to include non RCTs.  Type of deviation: Addition |
| Planned to only include outcome measures on bone mineral density and muscular function | Again, due to limited available research in this field following the initial review more primary outcomes such as quality of life, psychological well-being, disease activity, physical activity levels, body composition measures, cardiopulmonary measures, immunological outcomes and fatigue were included.  Type of deviation: Addition |
