# Supplementary material for: Effects of structured exercise programmes on physiological and psychological outcomes in adults with inflammatory bowel disease (IBD): A systematic review and meta-analysis
Source: PLoS One. 2022 Dec 1;17(12):e0278480. doi: 10.1371/journal.pone.0278480 (PMC9714897; doi:10.1371/journal.pone.0278480)
Supplement: S2 Table — (DOCX) [file pone.0278480.s003.docx]

**S2 Table.** Ongoing trials

| **Table 1**. List of ongoing trials of interest | | | |
| --- | --- | --- | --- |
| NCT Number | Status | Study Description | Estimated Completion |
| ISRCTN10756924: Physical training and nutrition before abdominal surgery for Crohn's disease | Recruiting*^a^* | It is hypothesised that an intensive, supervised 4-week prehabilitation programme in people undergoing bowel resection for Crohn’s disease is feasible and results in an improvement in physical function from baseline to the week before surgery | January 2024 |
| NCT05455762: The Efficacy of Exercise Therapy in Patients With Inflammatory Bowel Disease | Not yet recruiting | This study aims to determine the effect of 8 weeks of exercise on the quality of life and muscle strength of patients with IBD. Patients will be randomized into 1) Intervention group: doing aerobic and resistance exercise according to physiotherapist prescription and 2) Control group: usual medical care. Both groups will continue their medications prescribed by the Gastroenterologist. After 8 weeks patients come back to the hospital for re-evaluation of measured indices. The primary outcome of this study is the patient quality of life assessed by the IBDQ questionnaire. Other outcomes that will be evaluated before and after 8 weeks are muscle strength measured by a dynamometer and laboratory markers such as ESR and CRP and tool calprotectin. Also, disease activity using partial mayo score and Harvey Bradshaw score will be calculated and compared before and after treatment. | November 2022 |
| NCT05174754: Response To Medical Therapy in Inflammatory Bowel Disease Patients Carrying-out a Prescribed Exercise Programme | Not yet recruiting | The investigators propose the use of a 20 week physician-derived exercise programme will lead to an improvement in physical fitness which will in turn lead to an increase in muscle mass, a reduction in visceral obesity resulting in an improvement in biologic response, disease biomarkers (including a reduction in circulating pro-inflammatory cytokines), fatigue scores and quality of life. | May 2023 |
| NCT04816812: The IBD-FITT Study - Moderate-intensity Exercise for IBD Patients With Moderate Disease Activity (IBD-FITT) | Recruiting*^a^* | The investigators aim to investigate whether exercise therapy during 12 weeks including a lesson on general healthy lifestyle in adult patients with a moderately active disease is more effective, compared to control patients only receiving a lesson on general healthy lifestyle recommendations. The three main categories of outcomes are 1) health-related quality of life, 2) general health status of the patients 3) explorative outcomes. The primary outcome is health-related quality of life, the secondary outcome is general health status measured by waist circumference, disease activity scores, blood pressure, and blood lipids, and the third outcome are explorative outcomes (none-disease specific quality of life scores, biomarkers of C-reactive protein, fecal calprotectin and immunology markers including interleukins). | December 2022 |
| NCT04589338: Impact of Different Types of Physical Activity in Patients With Chronic Inflammatory Bowel Disease (ACHILLE) | Recruiting*^a^* | This is a prospective interventional study. The investigators will include patients in clinical remission who are on stable treatment. These IBD patients will be followed to analyze the effects of physical activity on their inflammatory disease. Different exercises will be offered to two separate groups of randomly distributed patients: the first group will perform muscle building exercises while the other group will be offered aerobic exercises. There will also be a control group that does not initially benefit from a specific physical activity program. However, this group will be integrated into one of the other two groups after the 10-week follow-up. All three groups will perform the same baseline tests. These will be of different types: physical tests, biological tests and quality of life questionnaire | February 2023 |
| NCT04303260: Special Physical Exercises as a Therapeutic Intervention for Inflammatory Bowel Disease | Recruiting*^a^* | A randomized-controlled-double blinded pilot study. Twenty Inflammatory Bowel Disease patients, suffering from mild disease will be randomized to undergo either a set of specific physical exercises for Inflammatory Bowel Disease or a control set of unrelated exercises. | December 2025 |
| NCT04143490: Effects of Exercise in Patients With Ulcerative Colitis | Suspended (Due to COVID-19) | The aim of this study is to investigate the effects of moderate to high intensity exercise on gut function and inflammatory markers in patients with Ulcerative Colitis (UC), and to compare them with individuals of matched age who do not suffer from Inflammatory Bowel Disease. Participants will be asked to complete 60 minutes of continuous cycling at a moderate to high intensity followed by a three hour recovery period. Gastrointestinal function will be measured using a non-invasive breath test. Blood samples will be collected at multiple time-points during the visit and these samples will be measured for markers of immune function and intestinal integrity | December 2021 |
| NCT02849717: Pre-Habilitation Exercise Intervention for Patients Scheduled for Colorectal Surgical Resection | Recruiting*^a^* | The proposed randomized controlled trial aims to recruit 60 patients that have a primary diagnosis of either colon or rectal cancer, inflammatory bowel disease, or diverticular disease and are scheduled for elective surgery. This two-arm clinical trial of an intervention examining the efficacy of a home-based walking and progressive resistance exercise program for the relief of post-operative fatigue and improvement of functional outcome following surgery | December 2023 |
| NCT03177044: Behavioural Treatment for Functional Bowel Symptoms in Inflammatory Bowel Disease | Active, not yet recruiting | The primary aim of the project is to investigate whether a behavioural training programme improves troublesome bowel symptoms, that people with inflammatory bowel disease continue to have, despite their disease being controlled by medication. The other aim is to determine if there are factors which influence how well the training programme works.  People attending an Inflammatory Bowel Disease clinic in a tertiary hospital, with bothersome bowel symptoms despite disease control, will be asked to join the study. This involves 2 to 6 sessions with a pelvic floor trained physiotherapist over a period of 6 months with further follow up at 12 months. | December 2021 |
| NCT04273399: High-impact Exercise in Adults With Crohn's Disease (IMPACT CD) | Unknown | The aim of this study is to assess the feasibility of high-impact exercise for improving markers of bone and muscle health in adults with Crohn's disease, and compare the effects of exercise with a group of healthy age and sex matched controls. | October 2021 |
| NCT02861053: Inflammatory Bowel Disease : Could a Regular Physical Activity Reduce Patients Fatigue ? | Unknown | Our hypothesis is that a regular moderate physical activity could improve physical performance during an effort et could reduce the chronic fatigue and improve their quality of life | April 2018 |
| *^a^* No results available | | | |
