# Supplementary material for: Effects of structured exercise programmes on physiological and psychological outcomes in adults with inflammatory bowel disease (IBD): A systematic review and meta-analysis
Source: PLoS One. 2022 Dec 1;17(12):e0278480. doi: 10.1371/journal.pone.0278480 (PMC9714897; doi:10.1371/journal.pone.0278480)
Supplement: S3 Table — (DOCX) [file pone.0278480.s004.docx]

**S3 Table.** Risk of bias for all outcomes

Cochrane RoB 2.0 tool domains: 1) Bias arising from the randomisation process; 2) Bias due to deviations from intended interventions; 3) Bias due to missing outcome data; 4) Bias in measurement of the outcome; 5) Bias in selection of the reported result; 6) Overall bias

**Outcome**: Disease Activity (e.g. Crohn’s Disease Activity Index, Harvey Bradshaw Index)

|  | **Domain** | | | | | |
| --- | --- | --- | --- | --- | --- | --- |
| **Study** | **1** | **2** | **3** | **4** | **5** | **6** |
| Ng et al (2007) | Some Concerns | Some Concerns | Low | Some Concerns | Some Concerns | Some Concerns |
| Klare et al (2015) | Low | Some Concerns | Low | Some Concerns | Some Concerns | Some Concerns |
| Cramer et al (2017) | Low | Some Concerns | Low | Some Concerns | Some Concerns | Some Concerns |
| Tew et al (2019) | Low | Some Concerns | Low | Some Concerns | Low | Some Concerns |
| Seeger et al (2020) | Low | Some Concerns | Low | Some Concerns | Some Concerns | Some Concerns |
| Jones et al (2020) | Low | Some Concerns | Low | Some Concerns | Some Concerns | Some Concerns |

**Outcome**: Disease Activity (e.g. faecal calprotectin)

|  | **Domain** | | | | | |
| --- | --- | --- | --- | --- | --- | --- |
| **Study** | **1** | **2** | **3** | **4** | **5** | **6** |
| Klare et al (2015) | Low | Some Concerns | Low | Low | Some Concerns | Some Concerns |
| Tew et al (2019) | Low | Some Concerns | Low | Low | Low | Some Concerns |
| Seeger et al (2020) | Low | Some Concerns | Low | Low | Some Concerns | Some Concerns |
| Jones et al (2020) | Low | Some Concerns | Low | Low | Some Concerns | Some Concerns |

**Outcome**: Quality of Life/ Health-Related Quality of Life

|  | **Domain** | | | | | |
| --- | --- | --- | --- | --- | --- | --- |
| **Study** | **1** | **2** | **3** | **4** | **5** | **6** |
| Ng et al (2007) | Some Concerns | Some Concerns | Low | High | Some Concerns | High |
| Klare et al (2015) | Low | Some Concerns | Low | High | Some Concerns | High |
| Cramer et al (2017) | Low | Some Concerns | Low | High | Some Concerns | High |
| Tew et al (2019) | Low | Some Concerns | Low | High | Low | High |
| Seeger et al (2020) | Low | Some Concerns | Low | High | Some Concerns | High |
| Jones et al (2020) | Low | Some Concerns | Low | High | Some Concerns | High |

**Outcome**: Bone Health

|  | **Domain** | | | | | |
| --- | --- | --- | --- | --- | --- | --- |
| **Study** | **1** | **2** | **3** | **4** | **5** | **6** |
| Robinson et al 1998 | Low | Some Concerns | Low | Low | Some Concerns | Some Concerns |
| Jones et al 2020 | Low | Some Concerns | Low | Low | Some Concerns | Some Concerns |

**Outcome**: Muscular Function

|  | **Domain** | | | | | |
| --- | --- | --- | --- | --- | --- | --- |
| **Study** | **1** | **2** | **3** | **4** | **5** | **6** |
| Seeger et al (2020) | Low | Some Concerns | Low | High | Some Concerns | High |
| Jones et al (2020) | Low | Some Concerns | Low | Low | Some Concerns | Some Concerns |

**Outcome**: Psychological Well-Being

|  | **Domain** | | | | | |
| --- | --- | --- | --- | --- | --- | --- |
| **Study** | **1** | **2** | **3** | **4** | **5** | **6** |
| Ng et al (2007) | Some Concerns | Some Concerns | Low | High | Some Concerns | High |
| Sharma et al (2015) | Low | Some Concerns | Low | High | Some Concerns | High |
| Tew et al (2020) | Low | Some Concerns | Low | High | Low | High |

**Outcome**: Fatigue

|  | **Domain** | | | | | |
| --- | --- | --- | --- | --- | --- | --- |
| **Study** | **1** | **2** | **3** | **4** | **5** | **6** |
| Tew et al (2019) | Low | Some Concerns | Low | High | Low | High |
| Jones et al (2020) | Low | Some Concerns | Low | High | Some Concerns | High |

**Outcome**: Cardiorespiratory Fitness

|  | **Domain** | | | | | |
| --- | --- | --- | --- | --- | --- | --- |
| **Study** | **1** | **2** | **3** | **4** | **5** | **6** |
| Tew et al (2020) | Low | Some Concerns | Low | Low | Low | Some Concerns |

**Outcome**: Body Composition

|  | **Domain** | | | | | |
| --- | --- | --- | --- | --- | --- | --- |
| **Study** | **1** | **2** | **3** | **4** | **5** | **6** |
| Klare et al (2015) | Low | Some Concerns | Low | Low | Some Concerns | Some Concerns |
| Seeger et al (2020) | Low | Some Concerns | Low | Low | Some Concerns | Some Concerns |

**Outcome**: Immune Parameters

|  | **Domain** | | | | | |
| --- | --- | --- | --- | --- | --- | --- |
| **Study** | **1** | **2** | **3** | **4** | **5** | **6** |
| Sharma et al (2015) | Low | Some Concerns | Low | Low | Some Concerns | Some Concerns |
| Klare et al (2015) | Low | Some Concerns | Low | Low | Some Concerns | Some Concerns |

**Outcome**: Physical Activity

|  | **Domain** | | | | | |
| --- | --- | --- | --- | --- | --- | --- |
| **Study** | **1** | **2** | **3** | **4** | **5** | **6** |
| Ng et al (2007) | Some Concerns | Some Concerns | Low | High | Some Concerns | High |
| Tew et al (2019) | Low | Some Concerns | Low | High | Low | High |
| Seeger et al (2020) | Low | Some Concerns | Low | High | Some Concerns | High |
| Jones et al (2020) | Low | Some Concerns | Low | High | Some Concerns | High |

Cochrane ROB Crossover Trials tool domains: 1) Bias arising from the randomisation process; S) Risk of bias arising from period and carryover effects; 2) Bias due to deviations from intended interventions; 3) Bias due to missing outcome data; 4) Bias in measurement of the outcome; 5) Bias in selection of the reported result; 6) Overall bias

**Outcome**: Disease Activity (e.g. Crohn’s Disease Activity Index, Harvey Bradshaw Index)

|  | **Domain** | | | | | | |
| --- | --- | --- | --- | --- | --- | --- | --- |
| **Study** | **1** | **S** | **2** | **3** | **4** | **5** | **6** |
| Cronin et al (2019) | Low | Low | Some Concerns | Low | Some Concerns | Some Concerns | Some Concerns |

**Outcome**: Quality of Life/ Health-Related Quality of Life

|  | **Domain** | | | | | | |
| --- | --- | --- | --- | --- | --- | --- | --- |
| **Study** | **1** | **S** | **2** | **3** | **4** | **5** | **6** |
| Cronin et al (2019) | Low | Low | Some Concerns | Low | High | Some Concerns | High |

**Outcome**: Psychological Well-Being

|  | **Domain** | | | | | | |
| --- | --- | --- | --- | --- | --- | --- | --- |
| **Study** | **1** | **S** | **2** | **3** | **4** | **5** | **6** |
| Cronin et al (2019) | Low | Low | Some Concerns | Low | High | Some Concerns | High |

**Outcome**: Cardiorespiratory Fitness

|  | **Domain** | | | | | | |
| --- | --- | --- | --- | --- | --- | --- | --- |
| **Study** | **1** | **S** | **2** | **3** | **4** | **5** | **6** |
| Cronin et al (2019) | Low | Low | Some Concerns | Low | High | Some Concerns | High |

**Outcome**: Body Composition

|  | **Domain** | | | | | | |
| --- | --- | --- | --- | --- | --- | --- | --- |
| **Study** | **1** | **S** | **2** | **3** | **4** | **5** | **6** |
| Cronin et al (2019) | Low | Low | Some Concerns | Low | Low | Some Concerns | Some Concerns |

**Outcome**: Immune Parameters

|  | **Domain** | | | | | | |
| --- | --- | --- | --- | --- | --- | --- | --- |
| **Study** | **1** | **S** | **2** | **3** | **4** | **5** | **6** |
| Cronin et al (2019) | Low | Low | Some Concerns | Low | Low | Some Concerns | Some Concerns |

ROBINS-I Domains: 1) Bias due to confounding; 2) Bias in selection of participants into the study; 3) Bias in classification of interventions; 4) Bias due to deviations from intended interventions; 5) Bias due to missing data; 6) Bias in measurement of outcomes; 7) Bias in selection of the reported result; 8) Overall bias

**Outcome**: Disease Activity

|  | **Domain** | | | | | | | |
| --- | --- | --- | --- | --- | --- | --- | --- | --- |
| **Study** | **1** | **2** | **3** | **4** | **5** | **6** | **7** | **8** |
| Loudon et al (1999) | Moderate | Moderate | Low | NI | Moderate | Serious Risk | Moderate | Serious Risk |
| Candow et al (2002) | NI | NI | NI | NI | NI | Serious Risk | NI | Serious Risk |
| Fagan et al (2020) | NI | Moderate | NI | NI | NI | Serious Risk | NI | Serious Risk |
| Kaur et al (2022) | Serious Risk | Low | Low | NI | Low | Serious Risk | Moderate | Serious Risk |
| NI, No Information | | | | | | | | |

**Outcome**: Disease Activity: Inflammatory Markers

|  | **Domain** | | | | | | | |
| --- | --- | --- | --- | --- | --- | --- | --- | --- |
| **Study** | **1** | **2** | **3** | **4** | **5** | **6** | **7** | **8** |
| Fagan et al (2020) | NI | Moderate | NI | NI | NI | Moderate | NI | Moderate |
| NI, No Information | | | | | | | | |

**Outcome**: Quality of Life/ Health-Related Quality of Life

|  | **Domain** | | | | | | | |
| --- | --- | --- | --- | --- | --- | --- | --- | --- |
| **Study** | **1** | **2** | **3** | **4** | **5** | **6** | **7** | **8** |
| Loudon et al (1999) | Moderate | Moderate | Low | NI | Moderate | Serious Risk | Moderate | Serious Risk |
| De-Souza Tajiri et al (2014) | Serious Risk | Serious Risk | Moderate | NI | NI | Serious Risk | NI | Serious Risk |
| van Erp et al (2019) | Moderate | Low | Low | NI | Moderate | Serious Risk | Moderate | Serious Risk |
| Fagan et al (2020) | NI | Moderate | NI | NI | NI | Serious Risk | NI | Serious Risk |
| NI, No Information | | | | | | | | |

**Outcome**: Muscular Function

|  | **Domain** | | | | | | | |
| --- | --- | --- | --- | --- | --- | --- | --- | --- |
| **Study** | **1** | **2** | **3** | **4** | **5** | **6** | **7** | **8** |
| Candow et al (2002) | NI | NI | NI | NI | NI | Serious Risk | NI | Serious Risk |
| De-Souza Tajiri et al (2014) | Serious Risk | Serious Risk | Moderate | NI | NI | Serious Risk | NI | Serious Risk |
| NI, No Information | | | | | | | | |

**Outcome**: Psychological Well-Being

|  | **Domain** | | | | | | | |
| --- | --- | --- | --- | --- | --- | --- | --- | --- |
| **Study** | **1** | **2** | **3** | **4** | **5** | **6** | **7** | **8** |
| Loudon et al (1999) | Moderate | Moderate | Low | NI | Moderate | Serious Risk | Moderate | Serious Risk |
| Fagan et al (2020) | NI | Moderate | NI | NI | NI | Serious Risk | NI | Serious Risk |
| Kaur et al (2022) | Serious Risk | Low | Low | NI | Low | Serious Risk | Moderate | Serious Risk |
| NI, No Information | | | | | | | | |

**Outcome**: Fatigue

|  | **Domain** | | | | | | | |
| --- | --- | --- | --- | --- | --- | --- | --- | --- |
| **Study** | **1** | **2** | **3** | **4** | **5** | **6** | **7** | **8** |
| van Erp et al (2019) | Moderate | Low | Low | NI | Moderate | Serious Risk | Moderate | Serious Risk |
| Fagan et al (2020) | NI | Moderate | NI | NI | NI | Serious Risk | NI | Serious Risk |
| NI, No Information | | | | | | | | |

**Outcome**: Cardiorespiratory Fitness

|  | **Domain** | | | | | | | |
| --- | --- | --- | --- | --- | --- | --- | --- | --- |
| **Study** | **1** | **2** | **3** | **4** | **5** | **6** | **7** | **8** |
| Loudon et al (1999) | Moderate | Moderate | Low | NI | Moderate | Serious Risk | Moderate | Serious Risk |
| van Erp et al (2019) | Moderate | Low | Low | NI | Moderate | Serious Risk | Moderate | Serious Risk |
| NI, No Information | | | | | | | | |

**Outcome**: Body Composition

|  | **Domain** | | | | | | | |
| --- | --- | --- | --- | --- | --- | --- | --- | --- |
| **Study** | **1** | **2** | **3** | **4** | **5** | **6** | **7** | **8** |
| Loudon et al (1999) | Moderate | Moderate | Low | NI | Moderate | Moderate | Moderate | Moderate |
| van Erp et al (2019) | Moderate | Low | Low | NI | Moderate | Moderate | Moderate | Moderate |
| NI, No Information | | | | | | | | |

**Outcome**: Physical Activity

|  | **Domain** | | | | | | | |
| --- | --- | --- | --- | --- | --- | --- | --- | --- |
| **Study** | **1** | **2** | **3** | **4** | **5** | **6** | **7** | **8** |
| Fagan et al (2020) | NI | Moderate | NI | NI | NI | Serious Risk | NI | Serious Risk |
| NI, No Information | | | | | | | | |
