# Supplementary material for: Effects of structured exercise programmes on physiological and psychological outcomes in adults with inflammatory bowel disease (IBD): A systematic review and meta-analysis
Source: PLoS One. 2022 Dec 1;17(12):e0278480. doi: 10.1371/journal.pone.0278480 (PMC9714897; doi:10.1371/journal.pone.0278480)
Supplement: S4 Table — (DOCX) [file pone.0278480.s005.docx]

| **S4 Table.** GRADE approach  **Table 2**. Certainty of evidence using the GRADE approach | | | | | | | | |
| --- | --- | --- | --- | --- | --- | --- | --- | --- |
| **Outcome** | **No. of Studies/ No of participants** | **Study Design Grade** | **Risk of bias/certainty**  (Downgrade 1 if ≥50% of domains rated as ‘some concerns’ or ‘high’ on risk of bias on Cochrane risk-of-bias tool version 2) | **Indirectness of evidence**  (Downgrade 1 if significant differences in PICO factors that may result in change of outcome) | **Heterogeneity or inconsistency of effect**  (Potential downgrade if wide variance of point estimates across studies; minimal/no overlap of CIs; wide (<2) PI or significant heterogeneity tests) | **Imprecision**  (Does the 95% CI cross the line of no effect? If yes, Downgrade 1.  If no, Is the OIS† reached? If no, Downgrade 1) | **Publication bias**^*^ | **Certainty of Evidence** |
| Disease Activity | 6 RCTs^18,21,22,36,37,40^  n= 282 | High | Some concerns or high risk >50% **downgrade 1** | No important indirectness = **no change** | No important inconsistency = **no change** | OIS was not met = **downgrade 1** | No evidence of publication bias= **no change** | Low  (⊙⊙○○) |
| Health-Related Quality of Life | 6 RCTs^18,21,22,36,37,40^  n= 307 | High | Some concerns or high risk >50% **downgrade 1** | No important indirectness = **no change** | No important inconsistency = **no change** | 95% CI includes 0. OIS is not met = **downgrade 2** | No evidence of publication bias= **no change** | Very Low  (⊙○○○) |
| Abbreviations: GRADE, Grades of Research, Assessment, Development and Evaluation; RCT, Randomised Controlled Trial; PICO, Patient, Intervention, Comparison and Outcome; CI, Confidence Interval; PI, Prediction interval; OIS, Optimal Information Size  †Where there was no power calculation for the outcome, the OIS required was presumed to be met if n≥400^23^  * Publication bias decision was based around the following considerations: study design and study size ^23^ | | | | | | | | |
