# Supplementary material for: Effects of structured exercise programmes on physiological and psychological outcomes in adults with inflammatory bowel disease (IBD): A systematic review and meta-analysis
Source: PLoS One. 2022 Dec 1;17(12):e0278480. doi: 10.1371/journal.pone.0278480 (PMC9714897; doi:10.1371/journal.pone.0278480)
Supplement: S1 File — (DOCX) [file pone.0278480.s006.docx]

**S1 File.** Types of outcome measures

1. Bone Health Outcomes**,** determined using validated standardised clinical measures (e.g. bone densitometry, ultrasound, calipers) considered in this review included but not limited to:

- Bone mineral content (BMC) (g) of total body
- Bone mineral density (BMD) (g/cm^2^) (areal and volumetric) of total body at the lumbar spine, femoral neck and/ or total hip
- Bone metabolism biomarkers including: bone alkaline phosphate (BAP) alkaline phosphatase (ALP), serum osteocalcin (OC), albumin,

carboxy-terminal collagen type 1 crosslinks (CTX), N-telpoptide collagen type 1 cross linked (NTX), deoxypyridinoline (DPD), bone specific alkaline phosphate (BSAP), N-terminal propeptides of type I procollagen (P1NP), C-terminal propeptides of type I procollagen (C1NP), pyridinoline (PYD), bone sialoprotein (BSP) and isoform 5b of tartrate-resistant acid phosphatase.

- Fractures at any site
- Osteoporosis, osteopenia and osteomalacia

1. Muscle Health Outcomes, determined using validated standardised clinical tools (e.g. handgrip dynamometer, timed chair stands, isokinetic dynamometry, gait speed, timed up and go), considered in this review included but not limited to:

- Muscle mass of lower and upper extremities
- Muscle endurance of lower and upper extremities
- Muscle strength (N/kg_FFM_) of lower and upper extremities
- Muscle flexibility of lower and upper extremities
- Measures of physical performance

1. Quality of Life measures considered in this review included general measures of health-related quality of life determined using validated standardised clinical tools (e.g. Inflammatory Bowel Disease QOL Questionnaire, 36-item short form survey and EQ-5D-5L)
2. Psychological well-being measures, determined using validated standardised clinical tools (e.g. Hospital Anxiety and Depression Scale, PHQ-9, perceived stress scale and State Trait Anxiety Inventory) considered in this review included but not limited to:

- Depression
- Anxiety
- Stress

1. Disease Activity: determined using validated standardised clinical measures (e.g. faecal calprotectin, Crohn’s Disease Activity Index, Harvey Bradshaw Index, inflammatory cytokines and Clinical Activity Index)
2. Physical Activity Levels: determined using validated standardised clinical measures (e.g. accelerometer, pedometer and International Physical Activity Questionnaire)
3. Body Composition measures determined using validated standardised clinical tools (e.g. dual-energy x-ray densitometry, circumference and girth measures, bodpod, CT or MRI scans, bioelectrical impedance) considered in this review included but not limited to:

- Body mass index (kg/m^2^)
- Lean and fat mass (kg) (%)
- Skeletal mass (%) and muscle mass (%)
- Cross-sectional muscle area (mm^2^)
- Percentage body fat (%)
- Total body water (TBW) (lt, %), potassium (TBK), nitrogen (TBN)

1. Cardiopulmonary measures determined using validated standardised clinical tools (e.g. wingate test, exercise stress test, cardiopulmonary exercise test, submaximal treadmill test and incremental shuttle walk test) considered in this review included but not limited to:

- Maximal oxygen consumption (VO2 max) (mL.kg^-1^ min^-1^) and peak oxygen consumption (VO2 peak) (mL.kg^-1^ min^-1^)
- Peak aerobic and anaerobic mechanical power (Wpeak) (Watts.kg^-1^)
- Oxygen pulse (O2 pulse)
- Maximum heart rate (HRmax, beats/min), peak heart rate (beats/min), resting heart rate (beats/min)
- Maximum tidal volume (maxTv)
- Respiratory exchange ratio (RER)

1. Immunological outcomes determined using validated standardised clinical tools (e.g. endocrinological blood samples and immunotoxicity) considered in this review included but not limited to:

- Leukocytes,
- Lymphocytes
- Granulocytes
- Monocytes
- Neutrophils

1. Fatigue outcomes, determined using validated standardised clinical measures (e.g. Inflammatory Bowel Disease- Fatigue Scale, fatigue severity scale, multidimensional assessment of fatigue and blood tests such as ferratin and haemoglobin)

1. Safety: Adverse events

The proportion of participants who experience an adverse event, not necessarily caused by the intervention will be recorded. Adverse event outcomes that result in death, require hospitalisation, is life threatening, results in persistent or significant disability or incapacity. All other adverse events will be considered non-serious. Withdrawal due to an adverse event will be reported.
