# Supplementary material for: Effects of structured exercise programmes on physiological and psychological outcomes in adults with inflammatory bowel disease (IBD): A systematic review and meta-analysis
Source: PLoS One. 2022 Dec 1;17(12):e0278480. doi: 10.1371/journal.pone.0278480 (PMC9714897; doi:10.1371/journal.pone.0278480)
Supplement: S2 File — (DOCX) [file pone.0278480.s007.docx]

**S2 File.** Search strategy

**MEDLINE**

1. exp Crohn disease/ or crohn*.mp.
2. indeterminate colitis.mp.
3. (colitis and ulcerat*).mp.
4. ulcerative colitis.mp. or exp ulcerative colitis/ or colitis, ulcerative/
5. (inflammatory bowel disease* or IBD).mp.
6. or/1-5
7. exp exercise/
8. exp exercise therapy/
9. (exercise* or exercising).mp.
10. exp sports/
11. ((resist* or weight* or strength* or endurance or circuit* or aerobic* or cardio* or jump* or anaerobic or balance or interval or muscl* or isokinetic or isometric) adj2 training).mp.
12. (walking or running or sprinting or jogging or swimming or cycling or rowing or dancing or yoga or boxing or skipping or pilates or aqua).mp.
13. Or/7-12
14. 6 and 13
15. (exp animal/ or animal.hw. or nonhuman/) not (exp human/ or human cell/ or (human or humans).ti.)
16. 14 not 15
17. 16 [Limit to English Language]

**EMBASE**

1. exp Crohn disease/ or crohn*.mp.
2. indeterminate colitis.mp.
3. (colitis and ulcerat*).mp.
4. ulcerative colitis.mp. or exp ulcerative colitis/ or colitis, ulcerative/
5. (inflammatory bowel disease* or IBD).mp.
6. or/1-5
7. exp exercise/
8. exp exercise therapy/
9. (exercise* or exercising).mp.
10. exp sports/
11. ((resist* or weight* or strength* or endurance or circuit* or aerobic* or cardio* or jump* or anaerobic or balance or interval or muscl* or isokinetic or isometric) adj2 training).mp.
12. (walking or running or sprinting or jogging or swimming or cycling or rowing or dancing or yoga or boxing or skipping or pilates or aqua).mp.
13. Or/7-12
14. 6 and 13
15. (exp animal/ or animal.hw. or nonhuman/) not (exp human/ or human cell/ or (human or humans).ti.)
16. 14 not 15
17. 16 [Limit to English Language]

**CINAHL**

1. ((MH “inflammatory bowel diseases”) or (MH “colitis, ulcerative”) or (MH “crohn disease”))
2. TI ((inflammatory bowel disease or IBD or Crohn* or ulcerative colitis or colitis* or indeterminate colitis) OR AB (inflammatory bowel disease or IBD or Crohn* or ulcerative colitis or colitis* or indeterminate colitis))
3. 1 or 2
4. ((MH "exercise") or (MH “exercise therapy”) or (MH “sports”))
5. TI ((resist* or weight* or strength* or endurance or circuit* or aerobic* or cardio* or jump* or anaerobic or balance or interval or muscl* or isokinetic or isometric) N2 training) OR AB ((resist* or weight* or strength* or endurance or circuit* or aerobic* or cardio* or jump* or anaerobic or balance or interval or muscl* or isokinetic or isometric) N2 training)
6. TI (“walking” or “running” or “sprinting” or “jogging” or “swimming” or “cycling” or “rowing” or “dancing” or “yoga” or “boxing” or “skipping” or “pilates” or “aqua”) OR AB (“walking” or “running” or “sprinting” or “jogging” or “swimming” or “cycling” or “rowing” or “dancing” or “yoga” or “boxing” or “skipping” or “pilates” or “aqua”)
7. 4 or 5 or 6
8. 3 and 7
9. 8 [Limit to English Language]
10. 9 [Limit to Human]

**Cochrane Central Register of Controlled Trials (CENTRAL)**

1. MeSH descriptor: [inflammatory bowel disease] explode all trees
2. TI ((IBD or Crohn* or ulcerative colitis or indeterminate colitis) or AB (IBD or Crohn* or ulcerative colitis or indeterminate colitis))
3. #1 or #2
4. MeSH descriptor [exercise] explode all trees
5. MeSH descriptor [exercise therapy] explode all trees
6. MeSH descriptor [sports] explode all trees
7. ((resist* or weight* or strength* or endurance or circuit* or aerobic* or cardio* or jump* or anaerobic or balance or interval or muscl* or isokinetic or isometric) near/2 (training))
8. ‘walking’ or ‘running’ or ‘sprinting’ or ‘jogging’ or ‘swimming’ or ‘cycling’ or ‘rowing’ or ‘dancing’ or ‘yoga’ or ‘boxing’ or ‘skipping’ or ‘pilates’ or ‘aqua’
9. #4 or #5 or #6 or #7 or #8
10. #3 and #9

**SPORTDiscus**

1. “inflammatory bowel disease*”
2. TI ((inflammatory bowel disease or IBD or Crohn* or ulcerative colitis or colitis* or indeterminate colitis) OR AB (inflammatory bowel disease or IBD or Crohn* or ulcerative colitis or colitis* or indeterminate colitis))
3. 1 or 2
4. "exercis*" or “exercise therapy” or “sports”
5. TI ((resist* or weight* or strength* or endurance or circuit* or aerobic* or cardio* or jump* or anaerobic or balance or interval or muscl* or isokinetic or isometric) N2 training) OR AB ((resist* or weight* or strength* or endurance or circuit* or aerobic* or cardio* or jump* or anaerobic or balance or interval or muscl* or isokinetic or isometric) N2 training)
6. TI (“walking” or “running” or “sprinting” or “jogging” or “swimming” or “cycling” or “rowing” or “dancing” or “yoga” or “boxing” or “skipping” or “pilates” or “aqua”) OR AB (“walking” or “running” or “sprinting” or “jogging” or “swimming” or “cycling” or “rowing” or “dancing” or “yoga” or “boxing” or “skipping” or “pilates” or “aqua”)
7. 4 or 5 or 6
8. 3 and 7
9. 8 [Limit to English Language]
